# The revision of the pharmaceutical legislation — it is time to act for nuclear medicine in Europe

**DOI:** 10.1007/s00259-023-06472-1

**Published:** 2023-10-23

**Authors:** Marianne Patt, Clemens Decristoforo, Amélie de Martini, Michel Koole, Wim J. G. Oyen, Oliver C. Kiss

**Affiliations:** 1 Section Radiopharmacy, Department of Nuclear Medicine, University Hospital Augsburg, Augsburg, Germany; 2 Department of Nuclear Medicine, Medical University Innsbruck, Innsbruck, Austria; 3 European Association of Nuclear Medicine, Vienna, Austria; 4 Nuclear Medicine and Molecular Imaging, Department of Imaging and Pathology, KU Leuven, Louvain, Belgium; 5 Department of Biomedical Sciences and Humanitas Clinical and Research Centre, Department of Nuclear Medicine, Humanitas University, Milan, Italy; 6 Department of Radiology and Nuclear Medicine, Rijnstate Hospital, Arnhem, The Netherlands; 7 Department of Medical Imaging, Nuclear Medicine, Radboud University Medical Centre, Nijmegen, The Netherlands; 8 Department of Targetry, Target Chemistry and Radiopharmacy, Institute for Radiopharmaceutical Cancer Research, Helmholtz-Zentrum Dresden-Rossendorf (HZDR), Dresden, Germany

## Introduction

In the context of the reform of the European Union Pharmaceutical Framework, a very important legislation for the future of healthcare in the European Union, the European Association of Nuclear Medicine (EANM), has conducted regular advocacy efforts to bring specific aspects of nuclear medicine to the attention of the European decision-makers. This editorial aims at highlighting these aspects of the revision of the European Pharmaceutical Package that touch on the field of nuclear medicine.

The last decades have yielded tremendous advances in nuclear medicine with the advent of clinical positron emission tomography (PET) in the 1990s and the early 2000s [1], starting with the widespread use of [^18^F]FDG and further expansion by other PET radiopharmaceuticals and the clinical establishment of ^68^Ga-labelled somatostatin analogues in the mid-2000s [2], in combination with ^90^Y- and ^177^Lu-based analogues for targeted radionuclide therapy (RNT) [3], which finally lead to the approval of [^177^Lu]Lu-DOTATATE (Lutathera™) by EMA in 2017 and, of course, the tremendous clinical success of a number of PSMA-binding ligands for PET imaging and RNT of patients with prostate cancer, resulting in the recent EMA and FDA approval of [^177^Lu]Lu-PSMA-617 (Pluvicto™) [4].

All these developments were primarily driven by European academic institutes and clinical facilities within a regulatory environment defining these radiopharmaceuticals as medicinal products and therefore governing their use by the pharmaceutical legislation. The legal inclusion of radiopharmaceuticals into the pharmaceutical legislation was originally implemented in the Directive 89/343/EEC of 3 May 1989 [5] that also covered radionuclide generators, kits and radionuclide precursor and their authorisation requirements. The main points of this directive were incorporated into the current “Community Code Directive” Directive 2001/83/EC [6], which, together with regulation 726/2004/EC, has laid down the basic rules for manufacturing and marketing of medicinal products in the European Union for the last two decades. Despite several amendments, it has not undergone relevant changes in relation to radiopharmaceuticals since its release in 2001. At that time, nuclear medicine practice was dominated by the use of ^99^Mo/^99m^Tc-generators in combination with cold kits and by externally supplied, ready for use, radiopharmaceuticals. However, this situation has meanwhile changed considerably over the years with, e.g. increasing importance of in-house production [7], which currently is enabled by specific national exceptions from Directive 2001/83/EC, thus causing large heterogeneity in nuclear medicine practice and availability of radiopharmaceuticals throughout Europe. Despite the tremendous new developments of nuclear medicine having an irrefutable positive impact on the daily care of our patients and, specifically, the emerging successful applications of theranostics for both the diagnosis and treatment of cancer patients, radiopharmaceuticals were never in the focus of the European Commissions’ pharmaceutical strategy.

## History, from the pharmaceutical strategy to the pharmaceutical package: the EANM advocacy efforts

Boosted by the COVID-19 crisis and as part of a whole package of measures put in place to build a strong European Health Union in 2020, the European Commission published a communication on a Pharmaceutical Strategy for Europe which was announced as an ambitious long-term project in the area of health, “intended to make the European pharmaceutical system more patient-centred, future-proof and crisis-resistant” [8]. Overall goals were to create a suitable legal environment for innovation, secure supply, address shortages and contribute to the overall aims of Europe’s Beating Cancer Plan while maintaining high quality and safety standards, as well as reducing the overall environmental footprint, in summary an ambitious plan with well-justified rationales. Based on the above pillars, it included several legislative and non-legislative actions, with the main flagship action being the reform of the EU pharmaceutical legislation. The Pharmaceutical Strategy initially sets the goal to have a proposal for the revision of the EU pharmaceutical legislation ready in 2022. To succeed in this ambitious goal, the European Commission conducted a series of consultations and meetings to inform on the design of the reform and took account of the positions and priorities raised by stakeholders, including the EANM, interested parties and the general public.

A first public consultation in the second half of 2021 invited all stakeholders, interested parties and EU citizens to provide feedback via a questionnaire with the objective to evaluate the general pharmaceutical legislation and assess the impacts of possible changes in legislation. The EANM, as a scientifically orientated society, provided feedback to the questionnaire and took advantage of the possibility to upload a general statement emphasising the relevance of the in-house preparation of radiopharmaceuticals to address the special needs of nuclear medicine and its patients and to drive innovation. At that time, the EANM highlighted that the regulatory framework for in-house radiopharmaceuticals production is not harmonised throughout Europe and has resulted in unbalanced access to innovative radiopharmaceuticals based on national legislative particularities. The EANM therefore called for any future revision to consider the importance of in-house production of radiopharmaceuticals and ensuring quality and safety with harmonised standards and dedicated rules, such that the particular needs of nuclear medicine are taken into account.

In addition to EANM’s own reply to the consultation, the statement was shared with the nuclear medicine national societies across Europe who were encouraged to provide harmonised feedback to raise awareness at the European Commission level. These efforts were well received by the European Commission, resulting in nuclear medicine practitioners being identified as one of the top ten campaigns of the public consultation, mainly thanks to the efforts of the German nuclear medicine community [9].

Furthermore, the input of EANM to the consultation resulted in an invitation by the Directorate-General for Health and Food Safety, DG Santé (which is the directorate of the European legislative body in charge of developing the revised directive) to the EANM to provide advice on how to revise the Community Code Directive in order to address the special needs of nuclear medicine and specifically radiopharmaceuticals. The EANM gratefully accepted this invitation and provided detailed suggestions on the revision, including the specific suggestion to distinguish between the two major classes of radiopharmaceutical preparation types, i.e. those that make use of licenced kits and licenced radionuclides (kit-based radiopharmaceutical compounding) on the one hand and more complex radiopharmaceutical preparations on the other hand. This proposed differentiation was accompanied by the suggestion for a revision of the existing definitions of kit and radionuclide precursor in order to restrict the need for a manufacturing authorisation to those starting materials that are used for kit-based radiopharmaceutical compounding activities which, from a regulatory point of view, are (already in the currently applicable legal framework) to be treated differently than “ordinary manufacturing practices” carried out outside a hospital pharmacy (Article 7 of Dir 2001/83/EC).

Following this extensive consultation process, the European Commission published on April 26, 2023, the legislative proposal for the review of pharmaceutical regulations. The publication, which is to be considered the most extensive reform in the pharmaceutical sector in more than 20 years, was very much welcomed by the health community. However, certain passages of the new legislation have sparked public criticism. Notably for the EANM, despite the extensive contribution to the consultation process, the legislative proposal did not include the aforementioned suggestions provided by EANM, most likely because of timelines that were too restrictive to be achieved and because of the conflictual nature of the legislative dossier.

## EANM proposal/intention

Now, with the publication of the legislative proposal, the general public as well as all other stakeholders (industry, patients, national regulatory bodies, national scientific communities and EANM) is once again invited to comment and provide feedback. Since harmonised suggestions supported by many stakeholders will have by nature better chances to be recognised, EANM has gone to great lengths to align with many other important stakeholders such as national nuclear medicine societies, patient representations, European hospital pharmacists (EAHP) and industry represented by Nuclear Medicine Europe (NMEU), hoping that this joint advocacy effort will allow the nuclear medicine community to have a much stronger impact on the ongoing and upcoming debates by speaking with a unified and common voice.

*Three main topics have been identified by EANM*

1. As was stated above, developments in radiopharmacy within the last two decades have shown that the current definitions with regard to radiopharmaceutical preparations are no longer fully applicable as they were intended to cover the radiopharmaceutical practice when Directive 2001/83/EC came into force. In particular, the ascent of complex radiopharmaceutical preparations now leads to a higher availability of individual, patient-centred radiopharmaceuticals in healthcare establishments (in-house preparations), that cannot be served by large-scale manufacturers. To further improve the availability of these in-house prepared radiopharmaceuticals, EANM proposes to restrict the need for marketing authorisation solely to those radionuclide precursors and radionuclide generators that are used either in kit-based radiopharmaceutical compounding or directly as medicinal products and to embrace radionuclide precursors or radionuclide generators that are used in complex radiopharmaceutical preparations as starting materials. This would highly increase the availability of radionuclides and radiopharmaceuticals to radiopharmacy and nuclear medicine departments for the benefit of our patients [11].
2. The second topic is related to the very heterogeneous landscape of radiopharmaceutical preparations within Europe. Due to the short half-life of the radionuclides used, in-house radiopharmaceutical preparation is an essential practice for nuclear medicine. Radiopharmaceutical preparation has been based on pharmacy practice (commonly known as the *magistral and officinal* *formula*) in some member states, whereas in other member states pharmacy practice does not cover radiopharmaceuticals [12]. Depending on the member states, preparations are either carried out in “classical” hospital pharmacies or, in nuclear medicine departments, research institutes and other entities to accommodate the specificities of radiopharmaceuticals. This is most of the time covered by specific national legislation, originating from the current wording of the Directive 2001/83/EC. This heterogeneity has already been identified in the revision of the Clinical Trial Directive and within the new Clinical Trial Regulation No. 536/2014 [10], by including the exemption for manufacturing authorisation for the “Preparation of radiopharmaceuticals, if this process is carried out in hospitals, health centres or clinics, by pharmacists or other persons legally authorised in the Member State concerned to carry out such process, and if the radiopharmaceutical is intended for ‘in-house’ use” (introduced in 2014 and with effect from 2023). The EANM proposes a similar provision for the revision of Directive 2001/83/EC.
3. The Council Basic Safety Standards Directive 2013/59/Euratom (BSSD) [13] has introduced the requirement to individually plan treatment involving ionising radiation, which includes treatment with radiopharmaceuticals. The missing alignment of the Directive 2001/83/EC with this requirement in the BSS Directive has caused an unclear situation in member states and should be clarified with the revision. As such, the EANM was pleased to see the point (19) of the recital in the proposed revision of Directive 2001/83/EC, stating that the revised Pharmaceutical Legislation Directive should be without prejudice to any provisions of Council Directive 2013/59/Euratom. It is very clearly stated that for all aspects related to radiation protection, and to posology and administration of radiopharmaceuticals, the BSS Directive should prevail over the Pharmaceutical Legislation Directive. However, this key statement is only included in the Recital of the Directive, which does not have any binding consequences (only setting the rationale behind the legal text). This means and this is of the utmost importance, that in the amendment process, similar provisions are included in relevant articles. A typical example here is Article 4 of the revised Directive, stating that for any medicinal product, the Directive should always prevail, hence contradicting Recital (19). To align with the Recital, Article 4 should be transformed to clearly state that nothing in this Directive shall in any way derogate from Council Directive 2013/59/Euratom.

## A delicate negotiation, with potential long-lasting impact on nuclear medicine: “nothing is finalised until everything is finalised”

After the publication of the legislative proposal by the European Commission, it is now in the hands of the co-legislators (the European Parliament and the Council). Following the Ordinary Legislative Procedure, the European Parliament and the Council of the EU, as co-legislators, have been preparing their positions on the overhaul of the EU medicines legislation ahead of discussions that are expected to take between 2 and 3 years.

Indeed, the main characteristic of the ordinary legislative procedure is the adoption of legislation jointly and on an equal footing by Parliament and the Council. It starts with a legislative proposal from the Commission and consists of up to three readings, with the possibility for the co-legislators to agree on a joint text — and thereby conclude the procedure — at any reading. Due to translation issues (i.e. the texts of the proposals cannot be formally presented to the European Parliament and Council until they have been translated into all of the EU’s official languages), the Parliament and Council have started to examine in parallel the Commission’s proposal only in September 2023.

While they are examining in parallel the Commission’s proposal, it is up to the Parliament to act first, voting by a simple majority and on the basis of a report prepared by one of its committees, in most cases either amending the Commission’s proposal or adopting it without amendments. It will only after that the Parliament has adopted its position that the Council will decide to accept Parliament’s position, in which case the legislative act is adopted, or it may adopt a different position at first reading and communicate it to Parliament for a second reading.

In the European Parliament, the Committee on Environment, Public Health and Food Safety (ENVI) is responsible for the file, guided by Pernille Weiss (EPP, Denmark), the rapporteur for this Directive. Work within the European Parliament on the legislation reform package has officially started on September 20th when the ENVI Committee Members met in Brussels and opened both the Commission’s proposal for a new directive and regulation. Overall, the proposal to revamp the European Union’s regulatory framework for pharmaceuticals was welcomed, but Members the European Parliament (MEPs) did not refrain from criticising certain aspects. Now MEPs, with the support of the Rapporteurs and Shadow Rapporteurs, will prepare their amendments to the Commission’s proposal and present them by November 14th back to ENVI, aiming to reach a common position and move the discussion to the plenary.

Considering both the Commission’s delay in presenting the file in spring and the delay in delivering the translations of the proposal, as well as taking into account that these discussions are expected to be delicate and conflictual, it therefore seems unlikely that the file will be approved within this mandate. Indeed, with the European Parliament election coming up in 2024, this leaves little time for the legislative process to take shape before the elections for the new European Parliament are held in June next year.

When adopted in plenary by the European Parliament, and then approved by the Council, likely somewhere in 2024, the procedure will move to the implementation stage. For the Directive, member states will receive a guideline and timetable for the implementation of the intended outcomes. With regard to the regulation, most likely, implementing regulations will be needed to ensure uniform implementation.

Thus, in light of the upcoming European elections in 2024, the negotiation process may well continue into the next mandate. Furthermore, in addition to health being a competence of the member states, the extensive and sensitive nature of the file will likely lead to lengthy negotiations in the Council of the EU. Therefore, there is no clear timeline for the adoption of the file, but the process is likely to go on until 2026 or even beyond.

In terms of immediate next steps opened to all stakeholders, a feedback consultation has been opened until November 2023, with all comments being summarised by the European Commission and presented to the European Parliament and the Council to feed into the legislative debate. In addition, at the level of the European Parliament, all stakeholders have the possibility to bring comments and feedback and suggested amendments to MEPs involved in the dossier.

The EANM is currently in discussion with several MEPs in order to suggest some amendments that would further strengthen the radiopharmaceutical provisions within the pharmaceutical package. Likewise, the EANM is replying to the European Commission’s feedback consultation and is encouraging the nuclear medicine community to do the same. For the whole nuclear medicine community, it is time to act now on the national level as well!

This revised pharmaceutical package has the potential to substantially modify the way radiopharmaceuticals are prepared and delivered in the decades to come, so is the time for the nuclear medicine community to raise any challenges and concerns we might have, the EANM is welcoming comments and suggestions on this topic.

## Data Availability

Not applicable.
